# The human Smoothened inhibitor PF-04449913 induces exit from quiescence and loss of multipotent *Drosophila* hematopoietic progenitor cells

**DOI:** 10.18632/oncotarget.10879

**Published:** 2016-07-28

**Authors:** Giorgia Giordani, Marilena Barraco, Angela Giangrande, Giovanni Martinelli, Viviana Guadagnuolo, Giorgia Simonetti, Giovanni Perini, Roberto Bernardoni

**Affiliations:** ^1^ Department of Pharmacy and Biotechnology (FaBiT), University of Bologna, Bologna, Italy; ^2^ Institut de Génétique et de Biologie Moléculaire et Cellulaire, CNRS/INSERM/ULP 67404 Illkirch, France; ^3^ Department of Experimental, Diagnostic and Specialty Medicine (DIMES), Institute of Hematology “L. e A. Seràgnoli”, University of Bologna, Bologna, Italy; ^4^ Health Sciences and Technology - Interdepartmental Center for Industrial Research (HST-ICIR), University of Bologna, Ozzano Emilia, Italy; ^5^ Present address: Department of Biological Sciences, School of Applied Sciences, University of Huddersfield, Queensgate, Huddersfield, UK; ^6^ Present address: Institute of Hematology, “L e A Seràgnoli”, S. Orsola-Malpighi Hospital, Bologna, Italy

**Keywords:** PF-04449913, Smoothened inhibitor, leukemia, Drosophila, prohemocytes

## Abstract

The efficient treatment of hematological malignancies as Acute Myeloid Leukemia, myelofibrosis and Chronic Myeloid Leukemia, requires the elimination of cancer-initiating cells and the prevention of disease relapse through targeting pathways that stimulate generation and maintenance of these cells. In mammals, inhibition of Smoothened, the key mediator of the Hedgehog signaling pathway, reduces Chronic Myeloid Leukemia progression and propagation. These findings make Smo a candidate target to inhibit maintenance of leukemia-initiating cells. In *Drosophila melanogaster* the same pathway maintains the hematopoietic precursor cells of the lymph gland, the hematopoietic organ that develops in the larva. Using *Drosophila* as an *in vivo* model, we investigated the mode of action of PF-04449913, a small-molecule inhibitor of the human Smo protein. *Drosophila* larvae fed with PF-04449913 showed traits of altered hematopoietic homeostasis. These include the development of melanotic nodules, increase of circulating hemocytes, the size increase of the lymph gland and accelerated differentiation of blood cells likely due to the exit of multi-potent precursors from quiescence. Importantly, the Smo inhibition can lead to the complete loss of hematopoietic precursors. We conclude that PF-04449913 inhibits *Drosophila* Smo blocking the Hh signaling pathway and causing the loss of hematopoietic precursor cells. Interestingly, this is the effect expected in patients treated with PF-04449913: number decrease of cancer initiating cells in the bone marrow to reduce the risk of leukemia relapse. Altogether our results indicate that *Drosophila* comprises a model system for the *in vivo* study of molecules that target evolutionary conserved pathways implicated in human hematological malignancies.

## INTRODUCTION

**Figure 1:**
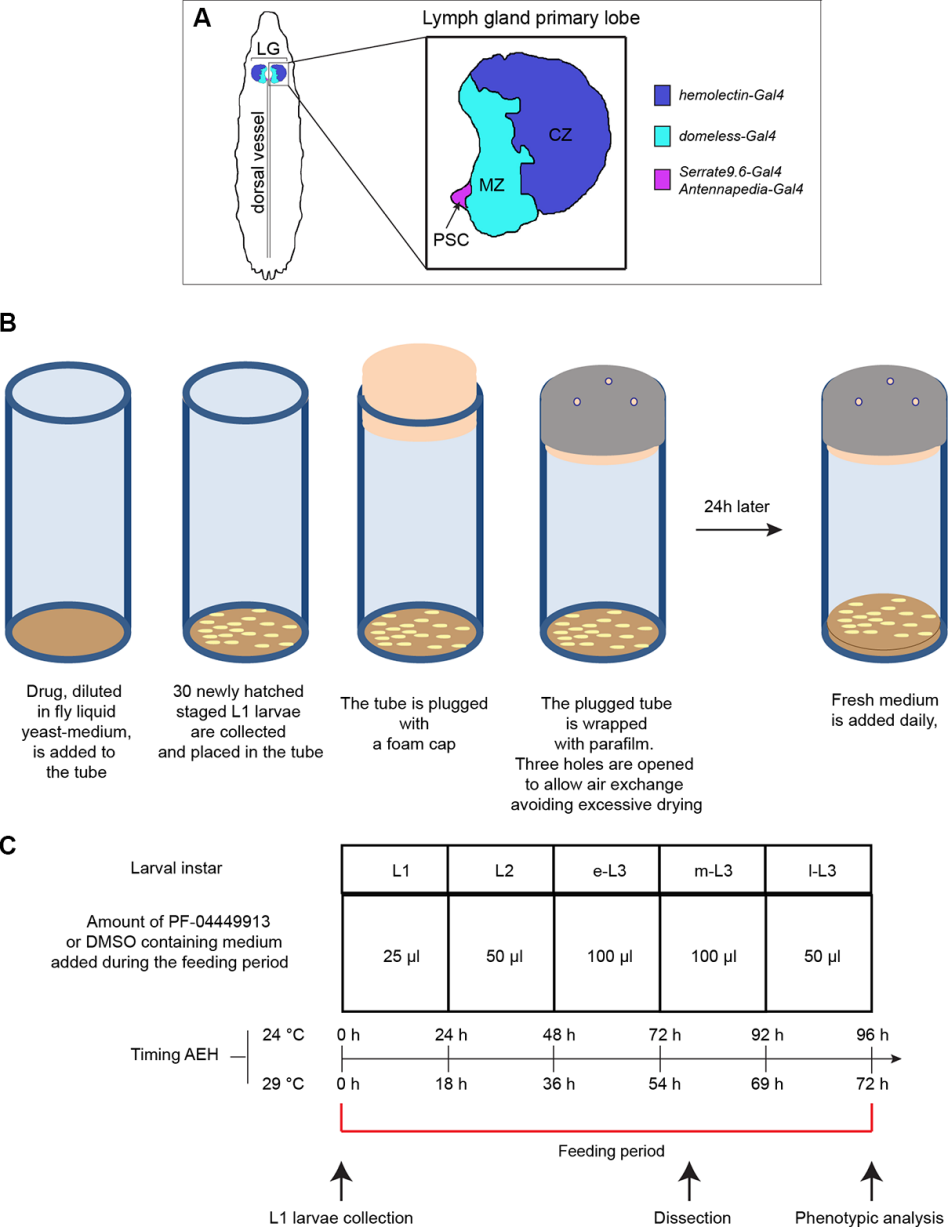
*Drosophila* lymph gland scheme and essential features of the drug administration protocol (**A**) Schematic drawing of a m-L3 lymph gland highlighting the position of the primary lobes (a primary lobe in the inset). MZ: medullary zone (green); CZ: cortical zone (blue); PSC: posterior signaling center (red). The color code indicates the lymph gland region in which are active the Gal4 trans-activating drivers used in the study. (**B**) Schematic drawings showing step-by-step the protocol followed for PF-04449913 or DMSO administration to newly hatched L1 larvae, collected and divided in batches of 30 animals each. (**C**) Table indicating: i) the amount of liquid yeast-medium containing PF-04449913 or DMSO added daily to each 30 animal batch; ii) the larval instars during the whole feeding period corresponding to each compound/DMSO administration; iii) the timing after embryo hatching (AEH) of the different developmental stages when animal are grown at 24°C or 29°C; iv) the time point at which larvae are collected, dissected or analyzed to evaluate the melanotic nodules penetrance and the larval feeding period are indicated. L1: 1st instar larva; L2: 2nd instar larva; e-L3: early 3rd instar larva; m-L3: mid 3rd instar larva; l-L3: late 3rd instar larva.

A key challenge in the treatment of solid tumor and hematological malignancies with targeted therapeutic agents is the induction of stable remission and attainment of functional cure. The persistence of the disease phenotype is attributable to the presence of minimal residual disease that can be maintained by Cancer Stem Cells (CSC) [1–3]. Examples of this are the myeloid malignancies, like Acute Myeloid Leukemia (AML), myelofibrosis (MF) and Chronic Myeloid Leukemia (CML). The phenotype of CML is driven by the constitutive kinase activity of the Bcr-Abl fusion protein [4, 5] and is effectively treated using first-, second- and third-generation tyrosine kinase inhibitors (TKIs) such as imatinib, dasatinib, nilotinib, bosutinib, and ponatinib that bind the ATP-binding site and, with varying degrees of selectivity, inhibit Bcr-Abl oncoprotein activity [6–10]. Although a percentage of patients successfully discontinue TKI therapy and achieve functional cure [11], in others leukemic stem/progenitor cells (LSCs) persist beyond treatment cessation and result in relapse [12–15]. The attainment of functional cure requires the identification of rational methods for targeting CSCs, which may be quiescent and out of cycle, as well as residual low frequency leukemic cells [16, 17]. In mice, loss of function of the seven-transmembrane domain protein Smoothened (Smo), a key component of the Hedgehog (Hh) signaling pathway, affects renewal of the hematopoietic stem cell (HSC) and inhibits LSC expansion reducing the transplantability of the disease in a CML murine model. Concurrent Bcr-Abl and Smo inhibition has been shown to eradicate LSCs in CML patients and murine models [18, 19]. PF-04449913 is a small-molecule inhibitor of the Smo protein, and is currently in clinical trials for the treatment of select myeloid malignancies [20]. A phase I safety and pharmacokinetics study suggested that treatment with PF-04449913 results in clinical responses in patients with AML, CML, myelodysplastic syndrome (MDS) and MF [21, 22]. The compound is thought to reduce maintenance of quiescent LSCs through the blockade of the signaling pathway activated by Hh ligands released from the bone marrow stroma. However, this has not been assessed *in vivo* in a hematopoietic tissue model that preserves the physiological contiguity of the different cell types. The dipteran *Drosophila melanogaster* has previously provided significant insights into oncogenic mechanisms. More recently, this well-established genetic model has been employed to study solid and hematological human malignancies, including their onset and progression. It has additionally been used in genetic screens and in the context of drug discovery platforms [23]. In a manner similar to vertebrates, *Drosophila* hematopoiesis is a developmental process that populates embryos, larvae and adults with circulating myeloid-like cells, known as hemocytes that regulate innate immunity and other processes. Three myeloid cell types circulate in the fly blood, the hemolymph: i) plasmatocytes, that are the most abundant, regulate the secretion of antimicrobial peptides and are provided with phagocytic ability to engulf small pathogens (i.e. bacteria) or cell debris; ii) crystal cells, that control the melanin production and the melanization process to inactivate big pathogen (i.e. eggs of parasite wasp); iii) lamellocytes, that are rarely seen in the hemolymph of healthy animals but that increase in number in case of big pathogen infestation since they are involved in the encapsulation of the pathogen that precedes melanization [24–26]. In the larva, part of the hemocytes circulates in the hemolymph and part resides at subepidermal locations (sessile hemocytes). In case of big pathogen infestation, i.e. wasp egg parasitism, this recently described sessile hemocyte compartment contributes most of the circulating blood cells activated by the immune response [27]. Under physiological conditions, the lymph gland (LG) is the hematopoietic organ that develops in the larva and breaks apart during metamorphosis to generate the hemocytes circulating in the adult hemolymph [24, 25, 28, 29]. The LG development begins in the embryo and in second instar larvae (L2) it grows by multipotent progenitor cells (prohemocytes) proliferation throughout the organ. In third instar larvae (L3), the LG is formed from three distinct functional regions: the medullary zone (MZ), populated by prohemocytes that at this stage rarely proliferate, suggesting that they enter a quiescence phase at the L2 to L3 transition; the cortical zone (CZ), made up of differentiating hemocytes and where most of the cells proliferating at this stage are concentrated; and the posterior signaling center (PSC) (Figure 1A). The latter behaves as a niche controlling blood progenitor cells maintenance through signaling pathways regulated by Notch, cytokines and Hh that are expressed by PSC cells and required for the maintenance of precursors located in the MZ (Figure 1A) [30–33]. Given that many transcription factors and signaling pathways involved in hematopoiesis are conserved between human and *Drosophila* [34, 35] and that Hh secreted from the PSC activates the Hh pathway in blood progenitors and contributes to their maintenance [31], we developed an *in vivo* genetic model based on *Drosophila* hematopoiesis to determine the impact at the cellular level of the small molecule PF-04449913, an inhibitor of the human Smo. Here we show that PF-04449913 impacts fly hematopoietic homeostasis, leading to increased and premature hemocyte differentiation and to the formation of melanotic nodules. The appearance of these nodules results from an excess of circulating lamellocytes and most likely to the formation of cell aggregates that are encapsulated and inactivated by the melanization process. They provide an easy-to-score phenotype due to the associated excess of blood cells and lamellocyte in particular [28, 36, 37]. Furthermore, the accessibility of the fly hematopoietic organ and the preservation of the proximity between the various cell types after the dissection procedure, made possible to show that PF-04449913 leads to accelerated hemocyte differentiation, lymph gland maturation and to CZ size increase at the expense of the multipotent precursor cells. Taken together, these results suggest that pharmacological inhibition of Smo in *Drosophila* alters hematopoietic homeostasis likely by inducing multipotent precursors to exit from quiescence, and accelerating blood cell differentiation. Moreover our body of evidence strongly supports *Drosophila* as a relatively cheap and fast genetic model to study and validate *in vivo*, at cell level, the way of action of small molecules targeting evolutionary conserved proteins and pathways implicated in hematological diseases and malignancies.

## RESULTS

### The Smo-inhibitor PF-04449913 impairs *Drosophila* blood cells homeostasis

**Figure 2:**
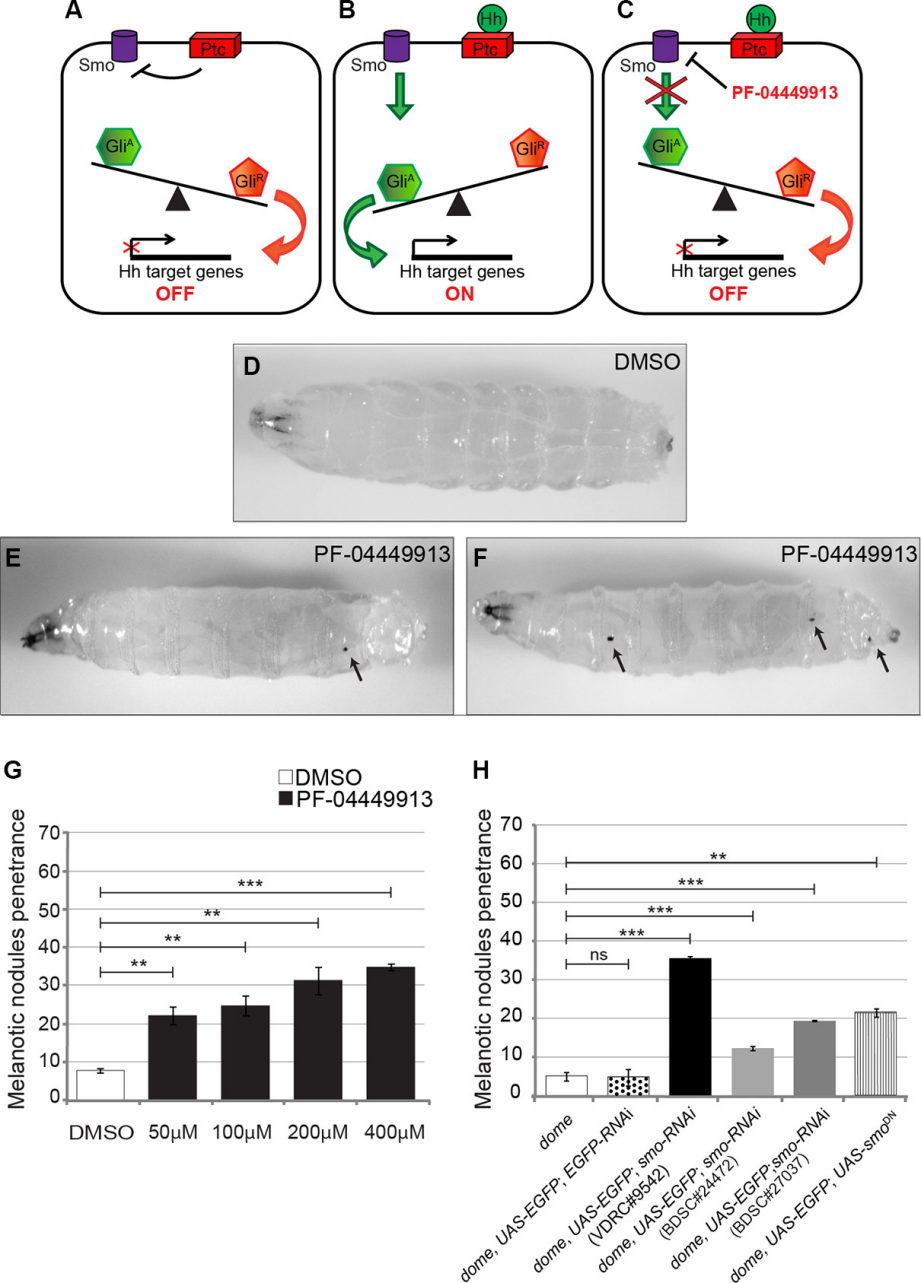
Pharmacological inhibition and genetic loss of the *smo* function alter blood cells homeostasis inducing melanotic nodules (**A**-**C**) Schematic drawings showing the expected consequences on the Hedgehog signaling pathway in the absence of Hh (A), in the presence of Hh (B) or after PF-04449913 administration (C). (**D**) A *w*^1118^ m-L3 instar larva raised on DMSO-containing medium (negative control). (**E**, **F**) *w*^1118^ m-L3 larvae raised on PF-04449913-containing medium. Black arrows in E and F indicate melanotic nodules. Anterior is on the left. (**G**) Penetrance of the melanotic nodule phenotype upon administration with increasing amounts of PF-04449913 to *w*^1118^ larvae compared to administration with DMSO. (**H**) Penetrance of the melanotic nodule phenotype due to the co-expression of EGFP with either one of three independent *smo*-RNAi constructs (VDRC#9542-black column, BDSC#24472-pale gray column, BDSC#27037-dark gray column) or of the dominant negative *smo*^5A^ construct (*smo*^DN^-vertical striped column) in the medullary zone (MZ) of the lymph gland under the control of the *domeless*-Gal4 driver. The negative controls, white and dotted columns, indicate, respectively, the penetrance in larvae carrying only the *domeless*-Gal4 driver (*dome*) or co-expressing EGFP and *EGFP*-RNAi under the control of *domeless*-Gal4 (*dome, UAS-EGFP*; *EGFP*-RNAi). The average phenotype penetrance due to compound administration was calculated from four to seven independent experiments, each involving 16–30 larvae. The average phenotype penetrance due to expression of either *smo*-RNAi or Smo^DN^ in the MZ was calculated from three to nine independent experiments, each involving 15–225 larvae. The statistic comparisons represent Student's *t* test (**P < 0.001, ***P < 0.0001, ns=not significant). Error bars indicate s.e.

**Figure 3:**
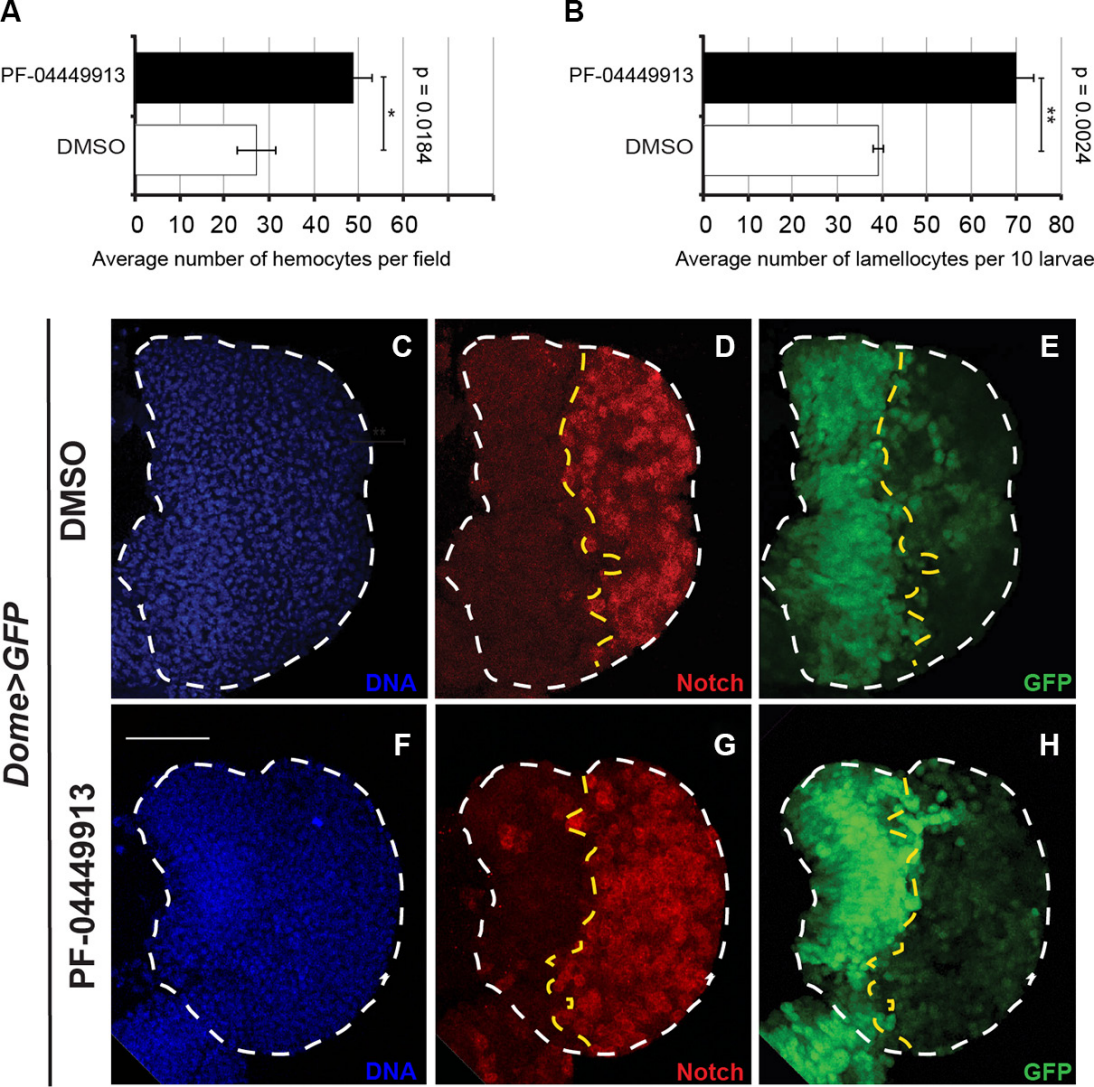
The human Smo inhibitor PF-04449913 drives the increase of hemocytes in the hemolymph and of crystal cells in the lymph gland cortical zone (**A**, **B**) Evaluation of the average number of hemocytes per field (A) and the total number of lamellocytes (ten larvae) (B) after bleeding of larvae fed with DMSO- or PF-04449913-containing medium. (**C**–**H**) Lymph glands from m-L3 larvae expressing EGFP under the control of the *domeless-Gal4* driver (*dome > EGFP)* administered with DMSO- (C-E) or PF-04449913- (F-H) containing medium. (C, F) nuclear Hoechst staining; (D, G) in red imunolabeling to detect the Notch receptor intracellular domain used as crystal cell marker; (E, H) EGFP expressed in the medullary zone. The statistic comparisons represent Student's *t* test (**P < 0.001, ***P < 0.0001, ns=not significant). Error bars indicate s.e. Scale bar: 100 μm.

Defining the mode of action of a drug at the cellular and molecular level is key to address its efficacy and specificity. In the *Drosophila* lymph gland, the absence of the PSC or the inhibition of the Hedgehog signaling pathway, reduces the number of multipotent hematopoietic precursors in the medullary zone and induces premature hemocytes differentiation [31]. To assess whether PF-04449913, an inhibitor of the human Smo protein (hSmo), antagonizes *Drosophila* Smoothened (Smo) activity and induces phenotypes that can be connected to loss of the Hh pathway function (Figure 2A–2C), we administered wild type (wt) animals with increasing concentrations of PF-04449913 (or equal amounts of DMSO-containing medium as a negative control) for the whole feeding phase of the larval life (Figure 1B, 1C). When compared with control larvae (Figure 2D), more animals fed with PF-04449913 showed 1 to 3 small melanotic nodules (Figure 2E, 2F) with dose-dependent penetrance. At all concentrations, the melanotic nodule penetrance was significantly higher than controls, peaking at 34% in larvae exposed to 400 μM PF-04449913 (Figure 2G). In order to determine whether decreased fly *smo* function in hematopoietic precursor cells induces melanotic nodules, we separately expressed one of three constructs (VDRC#9542, BDSC#24472, BDSC#27037) to produce small interfering RNAs targeting *smo* (*smo*-RNAi) or the dominant negative *smo*^5A^ allele (*smoDN)* [38] in the MZ under the control of the *domeless*-Gal4 (*dome*-Gal4) driver construct [39]. In each case, *smo* function knock-down or inhibition induced melanotic nodules with a penetrance ranging between 12.35% and 35.6%. The results were always significantly different to negative controls represented by larvae expressing GFP and *GFP*-RNAi under the control of the *domeless*-Gal4 driver or by larvae carrying the driver construct only (Figure 2H). The VDRC#9542 line was the most effective both in inducing melanotic nodules and in reducing Smo protein expression in the LG medullary zone as detected by immunolabeling (Supplementary Figure 1). All together these evidence suggest that PF-04449913 inhibits Smo activity. To assess whether the development of melanotic nodules correlates with increased numbers of differentiated cells in the hemolymph, we estimated the number of all circulating hemocytes and the number of lamellocytes. After feeding with PF-04449913 or DMSO-medium, we bled late-L3 (l-L3) instar larvae expressing GFP in all hemocytes under the control of the *hemolectin*-Gal4 *(hml*-Gal4*)* driver [33] and stained blood cells with Cy3 conjugated phalloidin. The *hemolectin*-Gal4 driver is active in differentiating hemocytes of the lymph gland CZ, in the circulating and sessile hemocytes. Phalloidin labels filamentous actin making easier to identify lamellocytes by their bigger size and flattened morphology [40]. Then, we counted GFP positive cells and lamellocytes. The average number of GFP positive hemocytes and lamellocytes were increased in larvae fed with 400 μM PF-04449913 as compared to those fed with DMSO (Figure 3A, 3B). This suggests that the melanotic nodules are correlated with the excess of circulating blood cells and in particular with the increase of lamellocytes, the hemocytes implicated in the encapsulation process that precedes melanisation [28, 36, 37]. During wasp egg infestation massive lamellocyte differentiation takes place. Most of the lamellocytes originates from the sessile hemocyte population that resides at subepidermal locations in a banded pattern. Only a part of the lamellocytes and circulating hemocytes have been shown to originate from the lymph gland [27]. To assess if the *domeless*-Gal4 driver is expressed in the sessile hemocytes population, we analyzed the GFP expression at the position where sessile hemocytes reside both in *dome-Gal4, UAS-GFP* and *hemolectin-Gal4, UAS-GFP* larvae. In *hml-Gal4, UAS-GFP* larvae we could clearly observe GFP expressing cells at the subepidermal positions where sessile hemocytes are localized [41] but we could not in *domeGal4, UAS-GFP* larvae (Supplementary Figure 2) We conclude that the *dome*–Gal4 driver is not active in the sessile hemocytes. This suggests that the melanotic nodule phenotype in *dome-Gal4, smo-RNAi* larvae should mostly depend on lymph gland derived hemocytes due to *smo* downregulation in the MZ. Although we cannot formally exclude that the hSmo inhibitor might act on hematopoietic compartments other than the lymph gland and induce an excess of circulating hemocytes, altogether our observations support the view that PF-04449913 should act on Smo expressing cells of the lymph gland MZ and induce increase of blood cell differentiation.

To evaluate if Smo inhibition can also induce increase of crystal cells differentiation, we dissected lymph glands from mid-L3 (m-L3) *dome-Gal4;UAS-GFP* larvae fed with PF-04449913- or DMSO-containing medium and labeled with an antibody that recognize the intracellular domain of the Notch membrane receptor. Notch is expressed and required in differentiated crystal cells of the CZ [42, 43]. Lymph glands from animals fed with the compound showed an increase of crystal cells scattered in the CZ compared to lymph glands from DMSO-fed animals (Figure 3C–3H). Taken together, these observations suggest that Smo maintains hematopoietic homeostasis in prohemocytes preventing their commitment to differentiation. This is consistent both with the role of the Hh signaling pathway in the MZ to maintain the quiescent prohemocytes [31] and with the formation of the melanotic nodules observed in larvae expressing *hh*-RNAi in the PSC cells under the control of either one of two different Gal4 drivers: *Serrate-9.6*-Gal4, and *Antennapedia*-Gal4 [31] (Supplementary Figure 3).

### PF-04449913 inhibits expression of an Hh signaling pathway target gene

**Figure 4:**
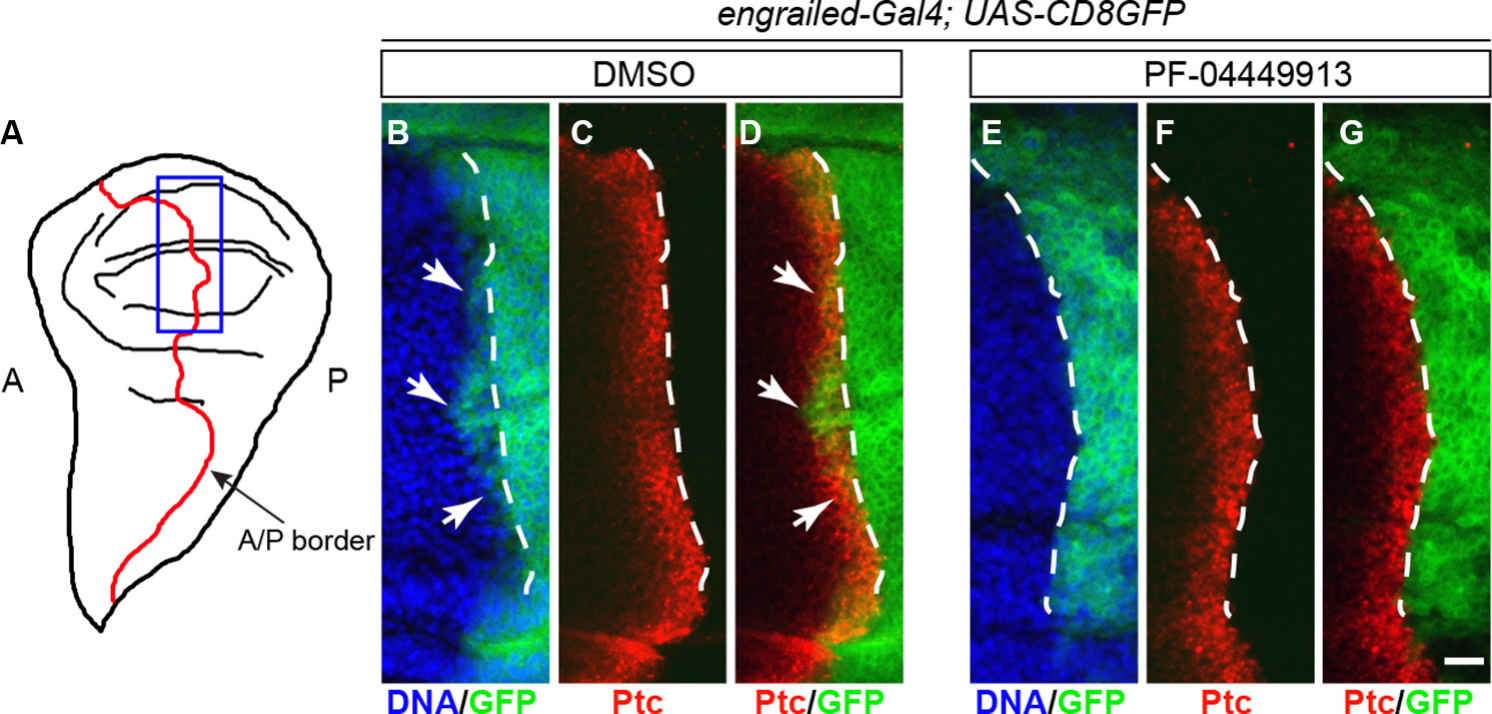
PF-04449913 inhibits expression of a Hh signaling target gene (**A**) Drawing of a wing imaginal disc in l-L3 larvae. The border (A/P border, red line) between the anterior-A and the posterior-P compartments is indicated. The blue rectangle indicates the region shown in the area of the imaginal discs magnified in B-G and corresponding to the A/P border in the presumptive wing blade, the wing pouch,. (**B**–**D**) A/P border of the wing pouch in a wing imaginal disc from an *engrailed-Gal4; UAS-CD8GFP* larva administered with DMSO-medium (negative control). (**E**–**G**) A/P border of the wing pouch in a wing imaginal disc from an *engrailed-Gal4; UAS-CD8GFP* larva administered with PF-04449913-medium. In green, the membrane-localized CD8-GFP chimera expressed under the control of the *engrailed*-Gal4 enhancer-trap line; in red, the immunolocalization of the Ptc protein; in blue, the cell nuclei stained by Hoechst. The white dashed lines indicate the A/P border as defined by the posterior limit of the high-level expression domain of the Ptc protein in the anterior compartment. White arrows indicate (B, D) the few cells of the anterior compartment expressing *engrailed* at low level under the control of the Hh signaling. *engrailed* expression in anterior cells is absent (E–G) in wing imaginal disc of larvae exposed to PF-04449913. Scale bar: 10 μm.

Since treatment of patients with PF-04449913 induces downregulation of the Hh signaling transcriptional target genes [22], we asked whether compound administration elicits downregulation of Hh positive target genes also in the *Drosophila* larvae. Although the role of Hh in the lymph gland is well described, the target genes of the Hh signaling pathway in this tissue have not been identified so far. However, the role of the Hh signaling is well established and described in the wing imaginal disc, the wing primordium that develops during the larval and pupal instars. In late L3 larvae this organ is formed by an anterior (A) and a posterior (P) cell compartment (Figure 4A) and the A/P border is well highlighted by the high level of expression of the Engrailed (En) transcriptional repressor in the whole P compartment. In the wing disc, Hh is expressed and secreted only in the posterior compartment where neither Patched (Ptc), the Hh membrane receptor, nor Cubitus interruptus (Ci), the transcriptional activator effector of the Hh signaling pathway, are expressed, since they are repressed by Engrailed. Ptc is expressed at low level, and independently from the Hh signaling pathway, throughout the anterior compartment. At the A/P border of the presumptive territory of the wing blade, the so called wing pouch, few anterior cell rows express Ptc at high level in a Hh dependent manner (Figure 4C, 4D) and the Hh signaling induces low level of En in few Ptc positive anterior cells abutting the A/P border [44–47] (Figure 4B, 4D). We adopted the Hh-dependent *ptc* and *en* gene expression in the anterior cells abutting the A/P border of the wing pouch as experimental paradigm to assess if PF-04449913 induces inhibition of Hh positive target genes. We fed with PF-04449913- or DMSO-medium larvae expressing a cell membrane-localized chimeric form of GFP under the control of the *engrailed*-Gal4 driver (*engrailed-Gal4*; *UAS-CD8GFP*) that drives Gal4 expression in the P compartment under the control of the *en* gene promoter. Wing imaginal discs were dissected from l-L3 larvae and immunolabeled with an antibody against the Ptc protein. In wing discs from DMSO treated larvae we could observe the high Ptc expression and the activation at low level of the *en*-dependent GFP-reporter in few anterior cells at the A/P border (Figure 4B–4D). In wing discs from larvae administered with PF-04449913 we could not observe any detectable change of the Ptc expression in the anterior cells at the A/P border. This might be due to the very high level of Ptc expression in these cells. On the other end, we could clearly note the total absence of the cell membrane localized GFP in anterior cells expressing high level of Ptc at the A/P border (Figure 4E–4G). This indicates that after PF-04449913 administration the Hh signaling does not activates the *engrailed*-Gal4 driver in the anterior cells, strongly supporting that PF-04449913 indeed inhibits the Hh-signaling dependent transcriptional program.

### PF-04449913 targets *Drosophila* Smo that is required to keep blood cell homeostasis when precursor cells enter quiescence

**Figure 5:**
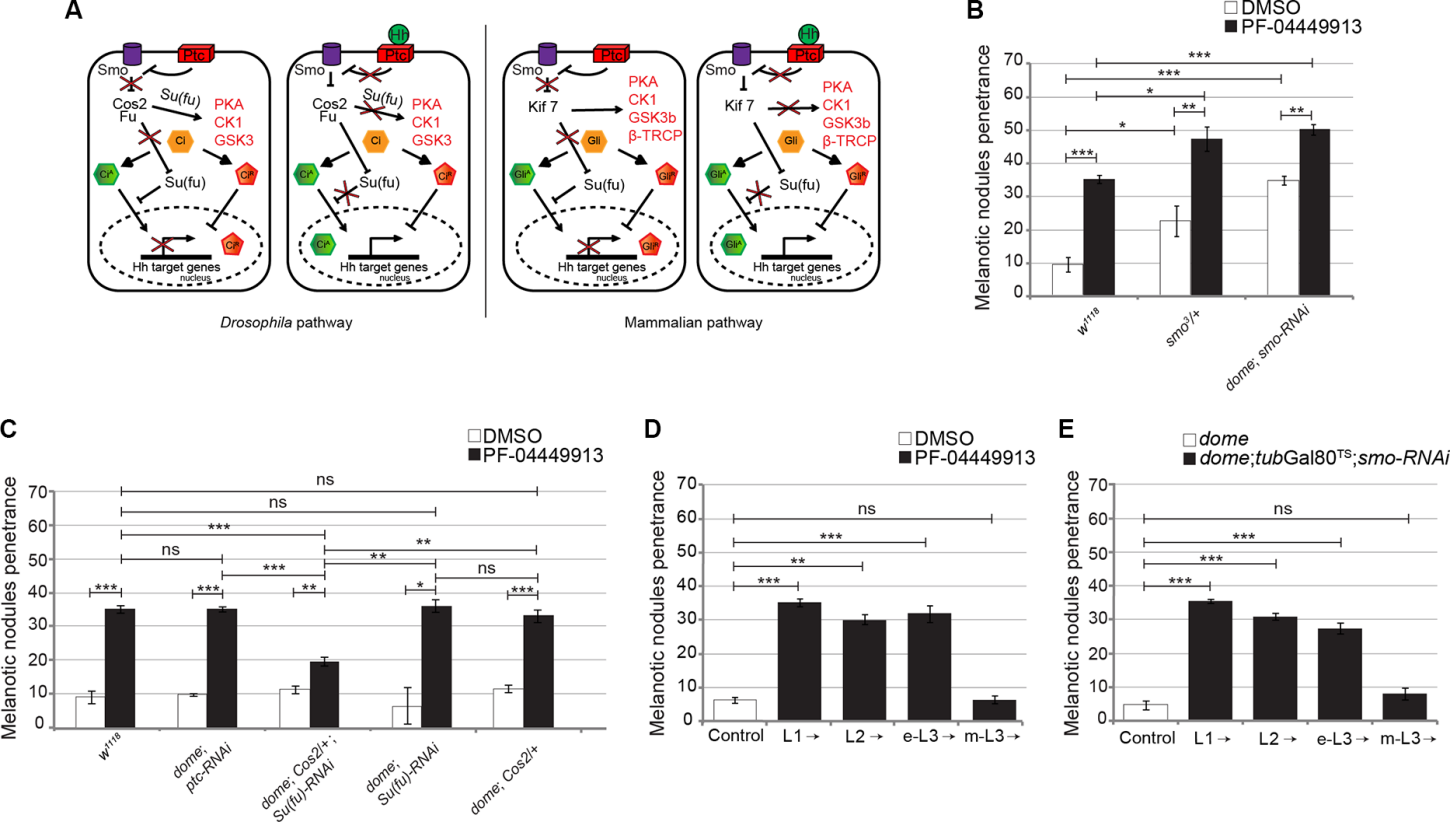
The PF-04449913 compound targets *Drosophila* Smo required to keep blood cell homeostasis at the time when precursor cells enter quiescence (**A**) Schematics comparing the function of key components of the *Drosophila* and mammals Hh signaling pathway and its transcriptional output in absence (left panels) or presence (right panels) of the Hedgehog ligand. (**B**) Penetrance of the melanotic nodule phenotype in *w*^1118^ (as wild type control) and *smo3/+* larvae or larvae expressing *smo*-RNAi in the MZ fed with PF-04449913- or DMSO-medium. (**C**) Penetrance of the melanotic nodule phenotype (PF-04449913 versus DMSO) in animals with constitutive activation of the Hh signaling pathway upstream (*ptc*-RNAi) or downstream (*dome; Cos*^2^*/+; Su(Fu)*-RNAi and the correspondent single controls: *dome; Cos*^2^*/+* and *dome; Su(Fu)*-RNAi) of Smo specifically in the MZ. (**D)** Penetrance of the melanotic nodule phenotype when PF-04449913 or DMSO feeding of wild-type larvae starts at the indicated developmental stages. Specific negative controls (DMSO) were performed for each time-point and the control histogram is referred to the average of these negative controls. (**E)** Penetrance of the melanotic nodule phenotype after conditional expression of *smo*-RNAi in the MZ (under the control of the *domeless*-Gal4 driver) and modulated by the *tubGal80*^TS^ system at the indicated developmental time points. The control column represents the average phenotypic penetrance of negative controls repeated at each experiment using animals carrying only the *domeless*-Gal4 driver. In panels B–E, the average phenotype penetrance is calculated from three to nine independent experiments, each involving 15–30 larvae. The statistic comparisons represent Student's *t* test (**P < 0.001, ***P < 0.0001, ns = not significant). Error bars indicate s.e.

To evaluate whether reduction of *smo* gene function is able to synergize with pharmacological inhibition, we fed larvae heterozygous for the *smo*^3^ null allele or expressing *smo*-RNAi in the MZ *(dome-Gal4;smo-RNAi)* with either DMSO- or PF-04449913-medium. Both reduction of *smo* gene dosage (*smo*^3^*/+)* and *smo* knock-down *(dome-Gal4;smo-RNAi)* induced melanotic nodules after DMSO-feeding in, respectively, 23% and 35.5% of larvae. This confirms the requirement of the Smo protein to maintain hemocytes homeostasis and indicates a partial aploinsufficiency of the *smo* gene function in hematopoiesis. The melanotic nodule penetrance increased to 47% and 50%, respectively, in *smo*^3^*/+* and *dome-Gal4;smo-RNAi* larvae, after administration of the compound (Figure 5B). Although *smo* function has been impaired combining either genetic LOF or tissue-specific RNAi and pharmacological inhibition, the phenotypic penetrance did not increase above 50%. This may result either from residual Smo function or from the activity of other signaling pathways controlling blood cells homeostasis [30, 33, 48]. To confirm that the hSmo inhibitor reduces Smo activity, we attempted to suppress the melanotic nodule phenotype by inducing constitutive activation of the evolutionary conserved Hh signaling pathway (Figure 5A). The binding of Hh to its receptor Ptc releases Smo from Ptc-mediated inhibition and prevents the three protein kinases PKA, CK1 and GSK3 from phosphorylating and degrading the transcriptional activator Ci that is maintained in the activator form (Ci^A^). In the absence of Hh: i) the three protein kinases form a complex with the scaffold kinesin protein Costal 2 (Cos2) and phosphorylate Ci leading to its proteolysis and conversion to the repressor form (Ci^R^); ii) the conformation of the Fused (Fu) kinase permits Suppressor of fused (Su(fu)) to impede Ci nuclear translocation and activity thereby promoting its conversion to the repressor form (Figure 5A) [49, 50]. We adopted established paradigms of constitutive activation of the *Drosophila* Hh pathway due either to LOF of Ptc, the regulator of the pathway upstream of Smo, or to concomitant LOF for two negative regulators of the pathway downstream of Smo, Cos2 and Su(fu) [47, 51–53]. We administered PF-04449913 to larvae expressing RNAi targeting the *ptc* function in the MZ (*dome-Gal4*;*ptc-RNAi*) or animals heterozygous for the *Cos2*^k16101^ null allele and expressing *Su(fu)*-RNAi in the MZ (*dome-Gal4;Cos2;Su(fu)-RNAi)*. While after compound administration *dome-Gal4;Cos2;Su(fu)-RNAi* larvae had a less penetrant melanotic nodule phenotype compared to the wt (19% vs 35%), the penetrance was unchanged in larvae expressing *ptc*-RNAi as well as in larvae either heterozygous for the *Cos2*^k16101^ allele or expressing *Su*(fu)-RNAi in the MZ (Figure 5C). The fact that the phenotype could be suppressed by constitutive activation of the pathway downstream of Smo but not by constitutive activation of the pathway upstream of Smo, strongly suggests that PF-04449913 acts on *Drosophila* Smo itself. To define when Smo is required to maintain blood homeostasis during development by preventing excessive blood cells differentiation, PF-04449913 administration or *smo*-RNAi conditional expression in the MZ was performed as from different time points during the larval feeding period (L1, L2, early L3:e-L3, m-L3) until the stage l-L3 when the phenotype was evaluated. Both approaches induced the phenotype when the pathway activation was inhibited as from L1, L2 and e-L3 but not when it was inhibited as from m-L3 (Figure 5D, 5E; for details see Supplementary Materials and Methods). This suggests that Smo is still required in multipotent precursors of e-L3 larvae, when the Hh signaling prevents hematopoietic precursor cells to undertake the pathway to differentiation. Indeed, the Hh protein is firstly expressed in PSC cells of L2 larvae and is required during the L3 instar to preserve in the lymph gland MZ, the pool of quiescent blood cell precursors expressing Smo and Ci [31, 33].

### PF-04449913 accelerates blood cells differentiation from hematopoietic precursors of the lymph gland medullary zone

**Figure 6:**
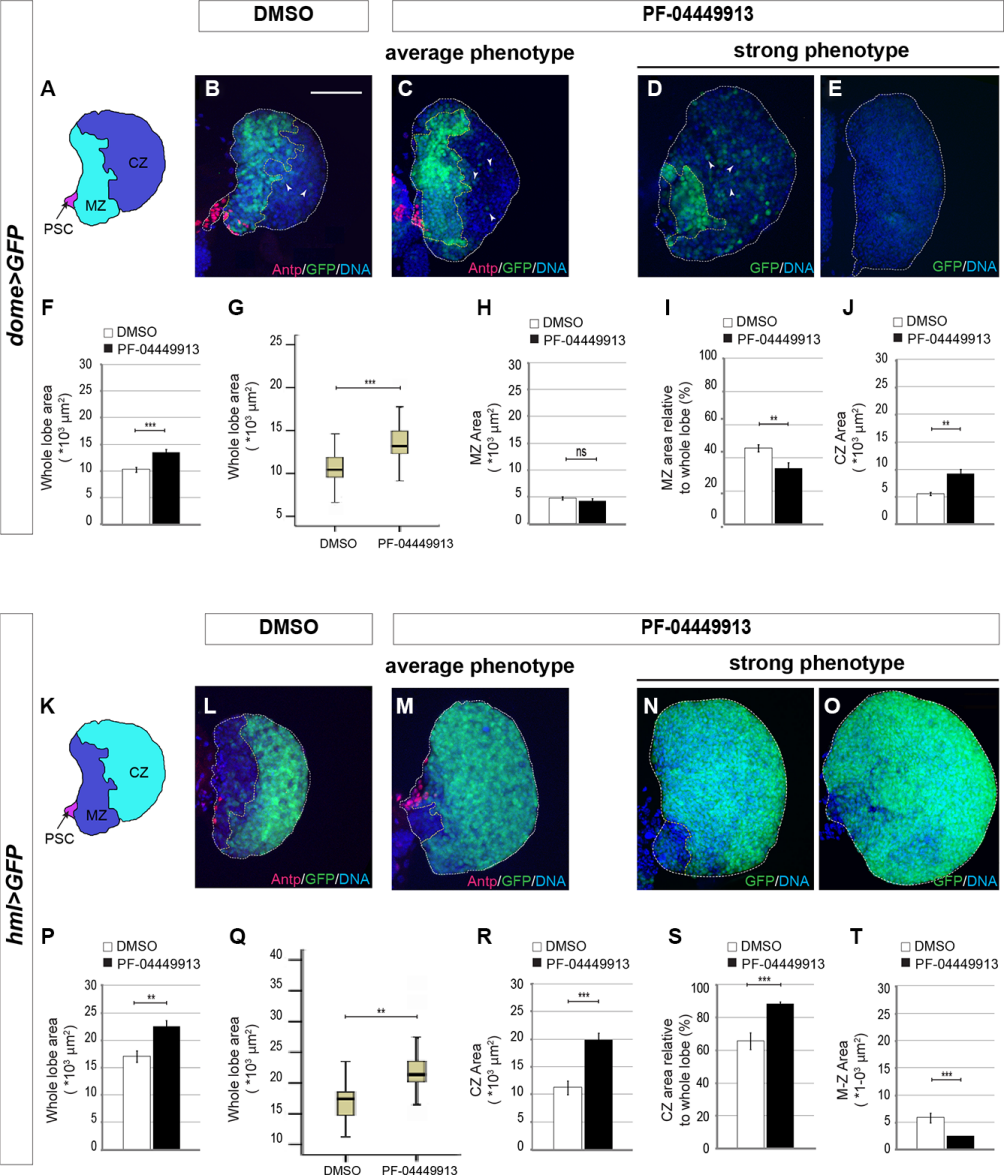
Smo inhibition upon PF-04449913 administration induces loss of hematopoietic precursor cells of the lymph gland medullary zone (**A**) Schematic drawing of a m-L3 lymph gland primary lobe. MZ: medullary zone (green); CZ: cortical zone (blue); PSC: posterior signaling center (red). (**B**–**E**) Lymph gland primary lobes from *domeGal4:UAS-GFP/+* m-L3 larvae (*dome* > GFP). Primary lobes are labeled by GFP in the hematopoietic precursors of the MZ, and by a DNA dye (blue). In B and C, lobes and MZ of average size from DMSO (B) and PF-04449913 treated animals (C) are labeled by an anti-Antp antibody to show the PSC cells (red). In D, E primary lobes showing a strong and incompletely penetrant phenotype in PF-04449913 treated larvae. (**F**–**J**) Quantification of the average whole primary lobe area (F), box plot showing the dispersion of the single values analyzed in F (G), absolute MZ area (H), MZ relative to whole lobe area (I), CZ area (J). White arrowheads in B–D indicate cells expressing low GFP level at the CZ position. (K) Schematic drawing of a m-L3 larval lymph gland primary lobe. MZ: medullary zone (blue); CZ: cortical zone (green); PSC: Posterior Signaling Center (red). (**L**–**O**) Lymph gland primary lobes from *hemolectin-Gal4:UAS-GFP/+* larvae (*hml* > GFP). Primary lobes are labeled by GFP in the differentiating hemocytes of the CZ, and by a DNA dye (blue). In L and M, lobes and CZ of average size from DMSO (L) and PF-04449913 treated animals (M) are also labeled by an anti-Antp antibody to show the PSC cells position (red). In N and O primary lobes displaying a strong and incompletely penetrant phenotype in PF-04449913 treated larvae. (**P**–**T**) Quantification of the average whole primary lobe area (P), box plot showing the dispersion of the single values analyzed in P (Q), absolute CZ area (R), CZ relative to whole lobe area (S), MZ area (T). For each condition the quantitative analysis is based on 18–21 primary lobes from 10–13 *dome* > GFP or *hml* > GFP larvae. All lymph glands are dissected from m-L3 larvae (75 h AEH at 24°C). Hoechst (blue) labels nuclei (DNA). Scale bar: 50 μm. All control lobes comes from larvae grown on DMSO-containing medium. The top and bottom along the Z-axis have been set to analyze the whole lobe. White columns correspond to control lobes, black columns show lobes from treated larvae. (**p < 0.001, ***p < 0.0001, ns = not significant). Error bars indicate s.e., except for the box plots in G and Q where the ends of the whiskers represent the minimum and the maximum value of the corresponding data.

The pharmacological inhibition of the Hh signaling induces an excess of circulating hemocytes. To assess whether this can be the consequence of increased commitment to differentiation of precursor cells of the MZ, we tightly synchronized and fed with PF-04449913-, or with DMSO-medium, hatching L1 larvae expressing GFP either in the MZ (*domeless-Gal4, UAS-GFP* - Figure 6A–6E), or in the CZ (*hemolectin-Gal4, UAS-GFP* - Figure 6K–6O). LGs from treated and control m-L3 larvae were dissected and analyzed to estimate the size of the GFP^+^ region (either MZ or CZ) and of the whole lymph gland primary lobe (for details see Supplementary Materials and Methods). We analyzed and compared both the average absolute size of GFP^+^ MZ, or CZ, and the relative size by calculating the average ratio between each MZ (or CZ) and the corresponding lobe size. In compound treated animals, LG primary lobes showed a modest but significant size increase compared to controls in both experimental sets both in term of average size change (Figure 6F, 6P) and in term of dispersion of the size values around the average (Figure 6G, 6Q). Unexpectedly, the average absolute size of the GFP^+^ MZ appeared unchanged (Figure 6H) and the relative MZ size decreased, accordingly with the modest but significant increase of the whole lobe size (Figure 6I). A possible explanation for that is the high stability of the GFP protein expressed under the control of the *domeless*-Gal4 driver that is active until such a time as the prohemocytes are committed to differentiation. However, due to protein stability, the GFP could persist in some differentiating cells and this leads to underestimate the loss of GFP^+^ MZ precursors in this experimental set. Our hypothesis is supported by the increase of scattered cells expressing low level of GFP in the CZ of *domeless-Gal4, UAS-GFP* larvae administered with the drug (Figure 6B–6D). Interestingly, both the absolute and the relative size of the GFP^+^ CZ of *hemolectin-Gal4, UAS-GFP* larvae fed with the compound significantly increased compared to controls (Figure 6R, 6S). Altogether these observations suggest that Smo inhibition induces the size increase of the LG primary lobe due to expansion of the CZ at the expense of the MZ as confirmed by the increase of the GFP negative region in *domeless-Gal4, UAS-GFP* larvae (Figure 6J) and by the decrease of the GFP negative region in *hemolectin-Gal4, UAS-GFP* larvae (Figure 6T). Similar results were obtained through co-expression in the MZ of GFP and a *smo*-RNAi construct (Supplementary Figure 4). Importantly, some lobes from *domeless-Gal4, UAS-GFP* treated larvae showed a significant reduction or even absence of GFP-expressing MZ cells (Figure 6D,6E) and, some lobes from *hemolectin-Gal4, UAS-GFP* larvae exposed to the compound were almost completely occupied by GFP expressing CZ cells (Figure 6N, 6O). This indicates that most or even all prohemocytes can be committed to differentiation after treatment with PF-04449913. This is consistent with the observation that in l-L3 larvae with melanotic nodules the lymph gland was very small, most likely because it has prematurely begun to break apart and to release the differentiated hemocytes (data not shown). In both experimental sets Antennapedia (Antp), that is expressed in PSC cells and required for their differentiation [31], is detected in a group of cells in the posterior part of the lymph gland primary lobe as observed by comparing immunolabeling of lymph glands between controls and treated animals (Figure 6A–6C, 6K–6M). This suggests that the decrease of prohemocytes could not be due to the absence of PSC cells [34]. To assess if the expansion of the CZ at the expense of the MZ is correlated to increase of cell proliferation in the lymph gland, we labeled lymph gland from *hemolectin-Gal4, UAS-GFP* larvae fed with PF-04449913 or DMSO with an antibody recognizing the phosphorylated histone H3 (PH3), a marker of cell proliferation. Then we counted PH3 positive nuclei of m-L3 lymph gland primary lobes on Z-stack projections of the max fluorescence intensity. We confirmed that Smo inhibition induces a significant increase of cell proliferation in the CZ of m-L3 larvae (Supplementary Figure 5). This supports the view that PF-04449913 may induce commitment of the MZ progenitor cells to undertake the proliferation and differentiation pathway determining the increase of the CZ and the increase of circulating hemocytes.

**Figure 7:**
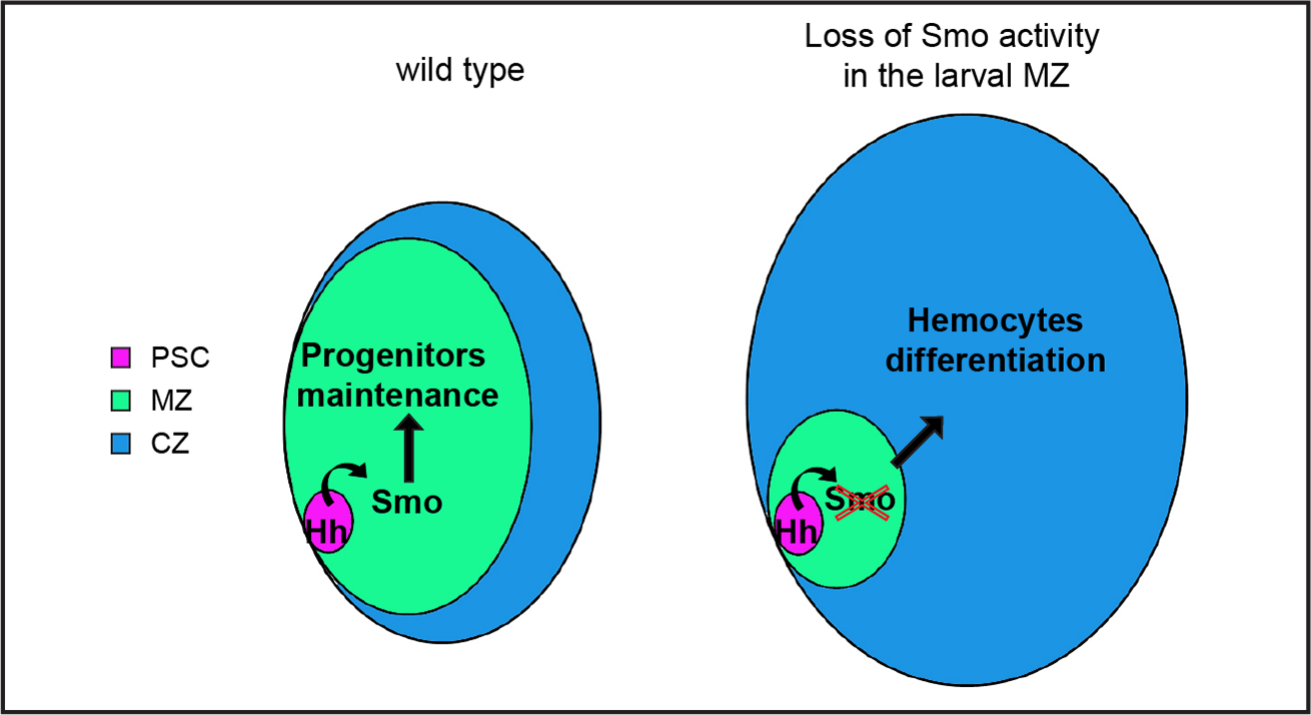
Scheme of lymph gland primary lobes showing the effect of Smo pharmacological inhibition upon PF-04449913 exposure or *smo* downregulation The different functional zones of the lymph gland primary lobe are indicated by different colors and the relative size changes describe that Smo functional inhibition decreases prohemocytes maintenance and increases differentiating hemocytes.

We conclude that the human Smo inhibitor PF-04449913 blocks the Hh signaling pathway activation in *Drosophila* hematopoietic precursor cells. This alters hematopoietic homeostasis most likely inducing multipotent precursor cells of the MZ to exit prematurely from quiescence and to undertake the differentiation pathway, migrate into the lymph gland CZ (Figure 7) and enter into the circulating hemolymph.

## DISCUSSION

One of the key issues in the development of drugs that target hematological malignancies as AML, CML and MF, is to devise therapeutic strategies that reduce the risk of disease recurrence. CML is a hematological malignancy in which functional cure may be obtained using compounds (TKIs) that target the singular pathogenic mechanism driven by the chimeric Bcr-Abl kinase. However, although tyrosine kinase inhibitors are effective at eradicating Bcr-Abl expressing cells, they do not routinely eliminate LSCs. These comprise a disease reservoir and lead to the development of TKI-resistant leukemic cells and subsequent relapse [12–15]. The elucidation of the mechanisms that control and maintain normal and pathological hematopoietic stem cells may provide a rational basis for new therapeutic targeting. A promising candidate pathway is the Hh signaling pathway. Smo, a key positive mediator of the pathway, regulates the self-renewal properties of murine HSCs as well as those of Bcr-Abl expressing LSCs in CML mouse models. Most importantly, Smo inactivation reduces the number of Bcr-Abl^+^ LSCs thereby inhibiting initiation and propagation of the disease in mice and reducing the expansion of LSCs from leukemia patients [18, 19]. The definition of a *Drosophila* model would be of significant utility in the study of new therapeutic compounds. *Drosophila* may be genetically manipulated and the hematopoietic organ, the lymph gland, is accessible for dissection and analysis, while also preserving the proximity between cell types. The lymph gland develops in the larva, and provides differentiated hemocytes that begin circulating at the onset of metamorphosis, but by the third larval instar following immune challenge or genetic manipulation. These conditions may lead to accelerated maturation of the lymph gland, increase of circulating cells, particularly lamellocytes, and to the development of melanotic nodules [28, 36]. The cells of the lymph gland PSC synthesize signaling molecules that control blood homeostasis through regulation of the maintenance of the multipotent precursor cells of the MZ. In L2 larvae, PSC cells begin to secrete Hh contributing to maintain quiescent precursor cells of the MZ that express the Hh receptor Ptc, Smo and the Hh pathway transcriptional effector Ci [31]. When new blood cells are required, the precursor cells divide to produce cells that migrate into the CZ, proliferate and differentiate [33, 54]. Thus, the functional conservation of the Hh signaling role in the hematopoietic process between mammals and *Drosophila* makes the latter an appropriate genetic model to analyze the effect of the hSmo inhibitor PF-04449913 *in vivo*. Wild-type larvae administered with increasing concentrations of this compound developed melanotic nodules at l-L3 with a dosage dependent penetrance. Moreover, larvae heterozygous for a *smo* null allele or expressing RNAi targeting *smo* specifically in multipotent precursors of the lymph gland MZ develop melanotic nodules. PF-04449913 administration indeed induced increase of differentiating crystal cells in the lymph gland CZ and of circulating total hemocytes and lamellocytes that might facilitate melanotic nodules development. Interestingly, while the constitutive activation of the Hh pathway through LOF of regulators acting downstream *smo* (*Cos2* and *Su(fu)*) could suppress the melanotic nodule phenotype induced by PF-04449913-mediated Smo inhibition, constitutive activation due to downregulation of *ptc,* that acts upstream *smo,* could not. Altogether these observations strongly suggest that the PF-04449913 compound inhibits *Drosophila* Smo that is expressed and required in the MZ to keep blood cells homeostasis. Reduction of the *smo* gene dosage or *smo* downregulation potentiated the compound effect, increasing the penetrance of the melanotic nodule phenotype up to 50%. Although residual Smo function may account for the incomplete phenotypic penetrance still observed in these animals after PF-04449913 feeding, this may also results from the action of other signaling pathways (including cytokine/JAK-STAT, Adenosine Deaminase Growth Factor A-Adgf A, Wingless/WNT, oxidative stress), active in the MZ and regulating blood cell homeostasis in response to different signals and environmental conditions [33, 48, 55–57]. Although it is not yet clear if these pathways play redundant functions or different roles in *Drosophila* blood cell precursors, our observations and the evolutionary conservations of the pathways suggests that the simultaneous inhibition of parallel pathways might more effectively prevent LSC maintenance and reduce the risk of leukemia relapse. This is confirmed by improved antitumor efficacy and survival observed in animal models following combined pharmacological inhibition of different pathways as those regulated by Hh and Notch among others [58]. Gene expression analysis on patients with hematological malignancies treated with the PF-04449913 has showed that the compound indeed downregulates Hh signaling positive target genes [22]. Interestingly, we could observe that in wing imaginal discs from PF-04449913 fed larvae the *engrailed* gene is not expressed in response to Hh signaling in few cells of the A compartment abutting the A/P border of the wing pouch. This is very similar to what has been observed in animals in which a LOF mutation of the *fused* gene blocked the Hh signaling [44]. We conclude that the PF-04449913 administration indeed inhibits Smo function and the transcriptional program regulated by the Hh signaling pathway.

The drug administration or the *smo*-RNAi expression at different stages of development showed that the e-L3 stage is the phenocritic phase, shortly after the PSC niche cells have begun to secrete the Hh ligand. Hh contributes to maintain the precursor cells of the lymph gland MZ that at this stage are quiescent or slowly cycling after the intense proliferative phase in L2 larvae [31, 33]. This suggests that Smo might contribute to the maintenance of the blood precursor population residing in the MZ. We confirmed this by reducing Smo function in animals that expressed GFP either in the MZ or in CZ cells. Lymph glands dissected from larvae treated with the compound, or larvae expressing *smo*-RNAi in the lymph gland MZ, showed that Smo inhibition results in loss of blood cell precursors and increase of differentiating hemocytes of the CZ that might enter the circulating hemolymph. This is concurrent with increased cell proliferation in the lymph gland, especially in the cortical zone, and is consistent with the tendency of the cortical zone to occupy almost the whole lymph gland primary lobe as documented by the huge expansion of the GFP expression domain in the differentiating hemocytes of the CZ. This is very similar to the expected effect of Smo inhibition by PF-04449913 in patients: the inhibition of one of the principal pathway controlling the quiescence and maintenance of normal and leukemic hematopoietic stem cells. The expected consequence is that the LSCs are forced to exit from the bone marrow and to enter the bloodstream where they become sensitive to therapeutic agents [18, 19, 21] such as TKIs in the case of Bcr-Abl dependent leukemias. In a mouse model of CML, Smo inhibition in hematopoietic progenitors transduced to express Bcr-Abl reduced the extent of disease development and increased disease latency in recipient animals, suggesting that the Smo protein may contribute to the maintenance of a reservoir of leukemia initiating cells [18, 19]. Using *Drosophila* as a simple model of hematopoiesis, we were able to directly observe in the hematopoietic organ the effect of pharmacological Smo inhibition and correlate it with the development of a phenotypic trait visible in living larvae. We observed that the human Smo inhibitor PF-04449913 or RNAi against *Drosophila smo* impairs hematopoietic homeostasis and induces the development of melanotic nodules. This phenotypic trait is associated to increase of hemocyte differentiation due to significant, and in some cases complete, loss of quiescent hematopoietic precursor cells. These features highlight the potential for using *Drosophila* as a model to facilitate the evaluation of the *in vivo* functional effectiveness of compounds targeting evolutionary conserved pathways involved in human hematological diseases.

## MATERIALS AND METHODS

### *Drosophila* growth conditions

Stocks and crosses were maintained at 24°C. Experimental progenies expressing transgenic constructs under the control of the Gal4/UAS system [59] were grown at 29°C. For experiments of tubulin-Gal80^TS^ mediated conditional Gal4/UAS dependent expression, crosses were maintained at the permissive temperature (18°C) and shifted to 29°C at the beginning of the stage of interest. Unless otherwise specified, larvae were analyzed for the melanotic nodule phenotype at late L3 instar. For further details see Supplementary Materials and Methods.

### Compound administration to *Drosophila* larvae, Gal4/UAS-mediated transgene expression and phenotypic assay

In order to establish control over the treatment timing and the concentration of compound administered to animals, we grew and treated the larvae in a defined volume of fresh yeast water suspension (liquid yeast-medium). PF-04449913 was dissolved in DMSO and diluted in the liquid yeast-medium to the indicated final concentration. Newly hatched and staged L1 larvae were harvested and divided into batches of 30 animals (Figure 1B). Since the compound shows a 24 h half-life [22] and may degrade when diluted in yeast medium, we added daily a defined amount of medium, with or without the compound, that could be completely consumed by the growing larvae (Figure 1C). For each trial at least three independent larvae batches were fed with either DMSO-containing food (control sample), or PF-04449913-containing food (experimental sample) at the indicated concentration, and allowed to grow at 24°C until l-L3 (96 h After Embryo Hatching-AEH) (Figure 1C). The feeding with the liquid yeast medium containing the compound or DMSO did not affect the growth since l-L3 larvae reached a normal size suggesting animals have eaten the medium with the compound or DMSO. We tested different concentrations in the range 0.5–10 μM without observing any obvious phenotype in larvae. Melanotic nodules and a weak effect on viability (survival rate around 90%) were observed in larvae exposed to 50–400 μM. Higher PF-04449913 concentrations (600–800 μM) were toxic. Crosses to produce animals with Gal4/UAS-mediated transgene expression were staged and grown at 29°C until l-L3 stage (Figure 1C). Harvested larvae were selected for the genotype of interest and analyzed for the melanotic nodule phenotype.

### Bleeding and preparation of hemolymph samples

Three groups of ten l-L3 instar larvae treated with PF-04449913 or DMSO and expressing GFP in all hemocytes under the control of the *hml*-Gal4 driver, were bled into 30 μl of *Drosophila* Ringer solution, labeled with Cy3 conjugated phalloidin (20 μg/ml, Sigma) that binds filamentous actin, and mounted under a coverslip with Fluormount. In order to evaluate the number of circulating hemocytes, GFP expressing cells were counted in three random fields per coverslip, and the average number of hemocytes per field between the coverslips was calculated. To evaluate the number of lamellocytes, we counted the phalloidin/GFP positive cells that showed big size and flat shape (compared to the small size and round shape of plasmatocyte and crystal cells) present under each coverslip and the average between samples was calculated.

### Lymph gland dissection, fluorescent immunolabeling and image analysis

Lymph glands from experimental and control larvae were dissected, fixed, and processed for fluorescent indirect immunolabeling with the indicated primary antibodies (see Supplementary Materials and Methods for details) and for DNA Hoechst staining. For each experimental or control group, 11–21 lymph gland primary lobes from 9–15 larvae were scanned through their whole thickness using a Nikon A1R confocal laser-scanning microscope, equipped with a Nikon PlanApo 40× lens and captured using the NIS Elements AR 3.10 software (Nikon). For each lobe, the GFP (corresponding to lymph gland MZ in *domeless-Gal4, UAS-GFP* larvae, or to CZ in *hemolectin-Gal4, UAS-GFP* larvae), the fluorescent signal generated by indirect immunolabeling and the Hoechst signal, were acquired along the Z-axis. The max fluorescence intensity of each Z stack was projected on a single image and the total lobe area (Hoechst) or the area occupied by GFP^+^ cells calculated using NIS Element software by outlining the area of interest. The total lobe area, the GFP^+^ area (MZ or CZ), the ratio between MZ (or CZ) over total area, and the difference between total area and the GFP^+^ area, were evaluated for each lobe and used to calculate the average and standard error (see Supplementary Materials and Methods for further details).

### Statistical analysis

Comparison between samples and statistical analysis was performed by applying the two-tailed Student's *t*-test to data from at least three independent experiments and calculating the *p*-value with the software GraphPad Prism 5. The software SPSS has been used to produce the Box Plots showed in Figure 6.

## SUPPLEMENTARY MATERIALS AND METHODS



## Abbreviations

AML: Acute Myeloid Leukemia
CML: Chronic Myeloid Leukemia
CSC: Cancer Stem Cells
TKI: tyrosine kinase inhibitor
LSC: leukemic stem cell
MDS: myelodysplastic syndrome
MF: myelofibrosis
LG: lymph gland
MZ: medullary zone
CZ: cortical zone
PSC: posterior signaling center
h: hour
AEH: After Embryo Hatching
GFP: Green Fluorescent Protein
EGFP: Enhanced Green Fluorescent Protein
DMSO: Dimethyl sulfoxide
Smo: Smoothened protein
*smo*: *smoothened* gene
Hh: Hedgehog protein
*hml*: *hemolectin*
wt: wild type
*dome*: *domeless*
*Ptc*: *Patched*
*Ci*: *Cubitus interruptus*
*Cos2*: *Costal 2*
*Su(fu)*: *Suppressor of fused*
*Antp*: *Antennapedia*
*Ser*: *Serrate*
*engrailed*: *en*
N^ICD^: Notch intracellular domain
LOF: Loss of function
A: anterior
P: posterior
L1: 1st instar larva
L2: 2nd instar larva
e-L3: early 3rd instar larva
m-L3: mid 3rd instar larva
l-L3: late 3rd instar larva
ns: not significant
s.e.: standard error
