# Supplementary material for: The human Smoothened inhibitor PF-04449913 induces exit from quiescence and loss of multipotent Drosophila hematopoietic progenitor cells
Source: Oncotarget. 2016 Jul 28;7(34):55313–27. doi: 10.18632/oncotarget.10879 (PMC5342419; doi:10.18632/oncotarget.10879)
Supplement: Supplementary file 1 [file oncotarget-07-55313-s001.pdf]

The human Smoothened inhibitor PF-04449913 induces exit from quiescence and loss of multipotent *Drosophila* hematopoietic progenitor cells

Supplementary Materials

SUPPLEMENTARY MATERIALS AND METHODS

Fly stocks and genetics

The following *Drosophila* stocks were used: w^{1118}, two *UAS-RNAi:smo* (*smo*-RNAi) lines (BDSC#27037 [1, 2], BDSC#24472 [3]), *UAS-smo*^{5A} *(UAS-smo*^{DN}*), smo*^{3}*, UAS-RNAi:ptc* (*ptc*-RNAi), *UAS-RNAi:Su(Fu)* (*Su(fu)*-RNAi), *UAS-RNAi:hh* (*hh*-RNAi) *Cos2*^{k16101}*, hemolectin-gal4, tubP-GAL80*^{TS}*, engrailed-Gal4* (BDSC#30564) and *5xUAS-CD8GFP* were obtained from the Bloomington *Drosophila* Stock Center (Bloomington, Indiana). w^{1118} flies were used as wild type. The *UAS-smo-RNAi* line #9542 was obtained from the Vienna *Drosophila* RNAi Center (VDRC) [4]. All the transgenic lines used to downregulate *smo* by RNAi express a 400–600 bp hairpin inverted repeat under the control of the Gal4 binding site UAS. The stocks *domeless-Gal4;UAS-GFP*, *Serrate9.6-Gal4* and *Antennapedia-Gal4* were, respectively, from S. Noselli [5, 6], U. Banerjee [7, 8] and M. Crozatier [9].

Drug administration to *Drosophila* larvae and phenotypic assay

Fertilized females of the different crosses were synchronized and let lay eggs for one hour. Newly hatched L1 larvae were collected, washed with water, and then divided in batches of 30 animals. The amount of either DMSO- or PF-04449913-containing medium has been increased day by day according to the progression of the larval instars of development: L1 25 µl, L2 50 µl, e-L3 100 µl, m-L3 100 µl. Then, larvae were collected and analyzed for the melanotic nodule phenotype at l-L3 or they were dissected at m-L3 and processed for immunofluorescence. Control larvae were identically handled to the ones exposed to drug. Each experiment was repeated at least three times and the shown data represent the average. Larvae were observed under a stereomicroscope to assess the presence of melanotic nodules. Since melanotic nodules may arise as a consequence of immune response against pathogens, for each trial and tested compound concentration, a concurrent DMSO control has been performed. The average phenotypic penetrance of control larvae ranged from 6% to 10%.

Gal4/UAS mediated expression

We set up appropriated and synchronized crosses at 29°C to induce expression of specific UAS transgenes under the control of the indicated Gal4 drivers. Progeny was allowed to grow until l-L3 (72 h AEH). Staged larvae were genotyped and selected according to the GFP fluorescence pattern of the constructs of interest and/or of the used GFP-balancer chromosomes. Larvae carrying the genotype of interest were observed under the stereomicroscope to assess the presence of melanotic nodules or were dissected to isolate the lymph gland then processed following the fixation/immunolabeling protocols

Lymph gland dissection and fluorescent immunolabeling

Lymph glands were dissected in PBS, kept on ice and then fixed for 30 minutes in 4% paraformaldehyde in PBS at room temperature. After 3 washes in PBS 1X pH 7.5, 0.3 % Triton (PBT 0.3%), lymph glands were rinsed for 30 minutes in PBT 0.3% and NGS 10% (Normal Goat Serum-Jackson Immunoresearch). The samples were incubated overnight at 4°C with the indicated primary antibodies in PBT 0.3% and NGS 10%. The day after the samples were washed three times in PBT 0.3% then incubated for 3 hours at room temperature with the secondary antibodies in PBT 0.3% - NGS 10% and the nuclei were labeled with Hoechst 33258 (Sigma) 2 µg/ml in PBS 1X. Tissues were mounted on slides under a coverslip using Fluormount. The anti-Smo mouse monoclonal antibody (supernatant diluted 1:300) developed by Beachy P.A [10, 11], the anti-Antp mouse monoclonal 4C3 (supernatant diluted 1:400; deposited to the DSHB by Brower D.), the anti-Ptc (Apa 1) mouse monoclonal antibody (concentrated Mab diluted 1:1000, deposited to the DSHB by Guerrero, I. (DSHB Hybridoma Product Drosophila Ptc (Apa 1)) and the anti-Notch mouse

monoclonal antibody raised against the Notch intracellular domain (N^{ICD}, diluted 1:1000, deposited to the DSHB by Artavanis-Tsakonas, S., DSHB Hybridoma Product C17.9C6) were obtained from the Developmental Studies Hybridoma Bank, created by the NICHD of the NIH and maintained at The University of Iowa, Department of Biology, Iowa City, IA 52242. The secondary antibody Goat-Cy3-anti-mouse (Jackson Immunoresearch) was diluted 1:400. The Rabbit anti-Phospho-histone H3 (PH3-Upstate Biotechnology, was diluted 1:100.

Imaging, quantification of the lymph gland phenotype

For each experimental or control group of animals, 11–21 lymph gland primary lobes have been scanned using a Nikon A1R confocal laser-scanning microscope, equipped with a Nikon PlanApo 40× lens and captured using NIS Elements AR 3.10 software (Nikon). For each lobe either GFP (corresponding to lymph gland MZ in *domeless-Gal4,UAS-GFP* larvae, or to CZ in *hemolectin-Gal4,UAS-GFP* larvae) or Hoechst (corresponding to the whole primary lobe) signals have been acquired along the Z-axis. The whole Hoechst signal was acquired along the Z-axis corresponding on average to 15.01 µm in control larvae and to 17.995 µm in larvae fed with the compound. The step among images was between 0.90 and 1.10 µm. Each Z stack has been projected on a single plane and the total lobe area (Hoechst) or the area occupied by GFP+ cells has been calculated using the NIS Element software.

Quantification of the proliferating cells

Lymph glands from m-L3 larvae fed either with PF-04449913- or DMSO-medium were dissected and immunolabeled with the anti-PH3 antibody and counterstained with the nuclear dye Hoechst. The whole thickness of 15 primary lobes from each group of larvae has been scanned using a Nikon A1R confocal laser-scanning microscope. Each Z stack has been projected on a single plane and the cells labeled bye anti-PH3 antibody has been counted.

REFERENCES

1. Ni JQ, Markstein M, Binari R, Pfeiffer B, Liu LP, Villalta C, Booker M, Perkins L, Perrimon N. Vector and parameters for targeted transgenic RNA interference in Drosophila melanogaster. Nat Methods. 2008; 5:49–51.
2. Kuzhandaivel A, Schultz SW, Alkhori L, Alenius M. Cilia-mediated hedgehog signaling in Drosophila. Cell Rep. 2014; 7:672–680.
3. Ogden SK, Casso DJ, Ascano M Jr, Yore MM, Kornberg TB, Robbins DJ. Smoothened regulates activator and repressor functions of Hedgehog signaling via two distinct mechanisms. J Biol Chem. 2006; 281:7237–7243.
4. Dietzl G, Chen D, Schnorrer F, Su KC, Barinova Y, Fellner M, Gasser B, Kinsey K, Oppel S, Scheiblauer S, Couto A, Marra V, Keleman K, et al. A genome-wide transgenic RNAi library for conditional gene inactivation in Drosophila. Nature. 2007; 448:151–156.
5. Bourbon HM, Gonzy-Treboul G, Peronnet F, Alin MF, Ardourel C, Benassayag C, Cribbs D, Deutsch J, Ferrer P, Haenlin M, Lepesant JA, Noselli S Vincent A. A P-insertion screen identifying novel X-linked essential genes in Drosophila. Mech Dev. 2002; 110(1–2):71–83.
6. Ghiglione C, Devergne O, Georgenthum E, Carballes F, Medioni C, Cerezo D, Noselli S. The Drosophila cytokine receptor Domeless controls border cell migration and epithelial polarization during oogenesis. Development. 2002; 129:5437–5447.
7. Jung SH, Evans CJ, Uemura C, Banerjee U. The Drosophila lymph gland as a developmental model of hematopoiesis. Development. 2005; 132:2521–2533.
8. Bachmann A, Knust E. Dissection of cis-regulatory elements of the Drosophila gene Serrate. Dev Genes Evol. 1998; 208:346–351.
9. Mandal L, Martinez-Agosto JA, Evans CJ, Hartenstein V and Banerjee U. A Hedgehog- and Antennapedia-dependent niche maintains Drosophila haematopoietic precursors. Nature. 2007; 446:320–324.
10. Lum L, Zhang C, Oh S, Mann RK, von Kessler DP, Taipale J, Weis-Garcia F, Gong R, Wang B, Beachy PA. Hedgehog Signal Transduction via Smoothened Association with a Cytoplasmic Complex Scaffolded by the Atypical Kinesin, Costal-2. Molecular Cell. 2003; 12:1261–1274.
11. Fan J, Liu Y, Jia J. Hh-induced Smoothened conformational switch is mediated by differential phosphorylation at its C-terminal tail in a dose- and position-dependent manner. Developmental Biology. 2012; 366:172–184.

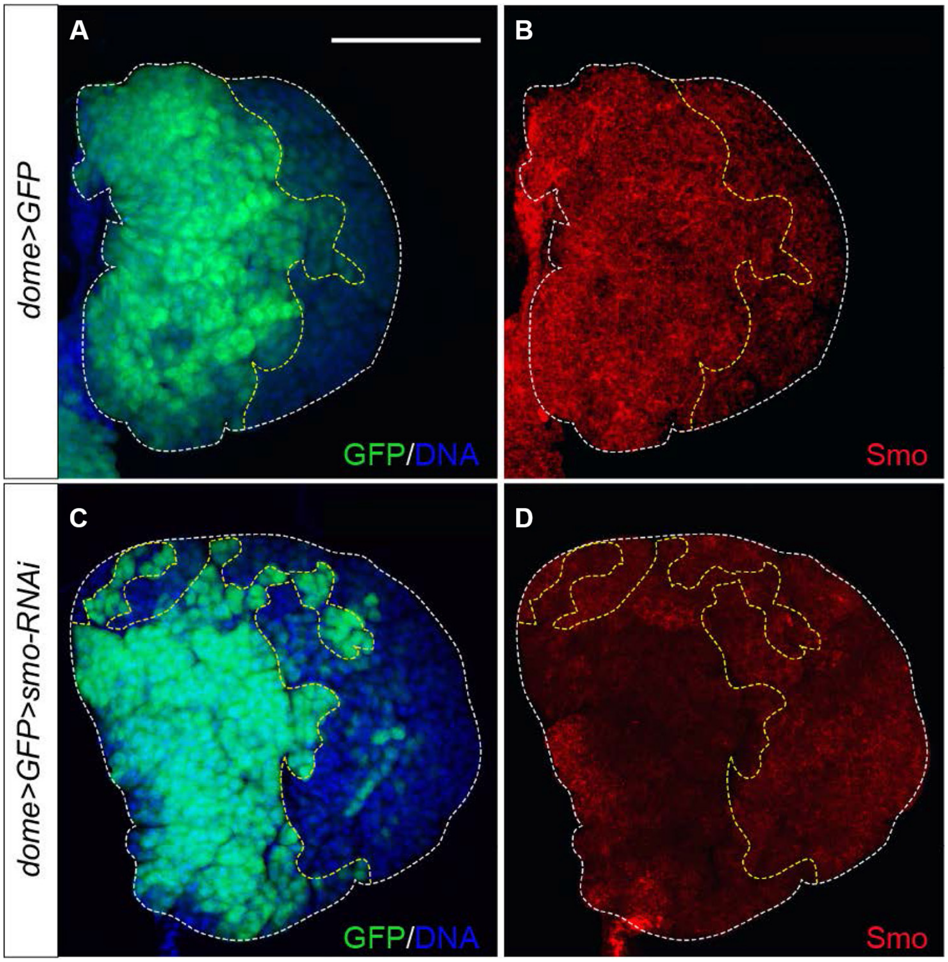

Supplementary Figure S1: *smo*-RNAi reduces dSmo protein levels in the hematopoietic precursor cells of the MZ. (**A**–**B**) Lymph gland primary lobes from *domeless-Gal4:UAS-GFP/+* m-L3 larvae (*dome* > GFP). Primary lobes are labeled by GFP in the hematopoietic precursors of the MZ, by an anti-Smo antibody (red) and by a DNA dye (blue). (**C**–**D**) Primary lobes from m-L3 larvae coexpressing GFP and *smo*-RNAi under the control of the *domeless-Gal4* driver (*dome* > GFP > *smo*-RNAi). The top and bottom along the Z-axis have been set to analyze the whole lobe. Scale bar: 50 µm.

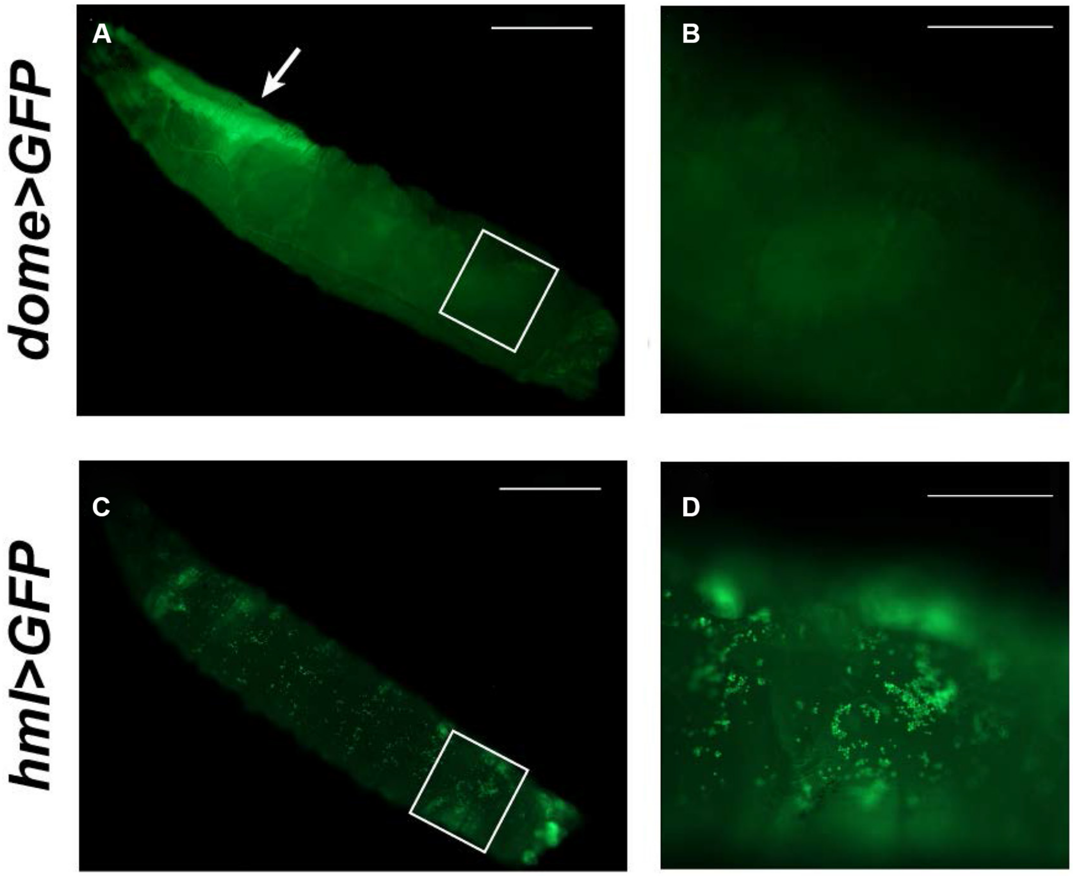

Supplementary Figure S2: The *domeless-Gal4* driver is not active in the sessile hemocytes. (**A**–**B**) A l-L3 d*omeless-Gal4,UAS-GFP (dome>GFP)* larva at low (A) or high (B) magnification. The white rectangle in A indicates the region highlighted in B and showing a position where sessile hemocytes accumulate at subepidermal position. The white arrow in A indicates high GFP expression driven by *domeless*-Gal4 in the salivary glands. (**C**–**D**) A l-L3 *hemolectin-Gal4,UAS-GFP (hml>GFP)* larva at low (C) or high (D) magnification. The white rectangle in C indicates the region highlighted in D and showing the sessile hemocytes accumulated at subepidermal positions. The scale bar in A and C corresponds to 500 µm; the scale bar in B and D corresponds to 250 µm.

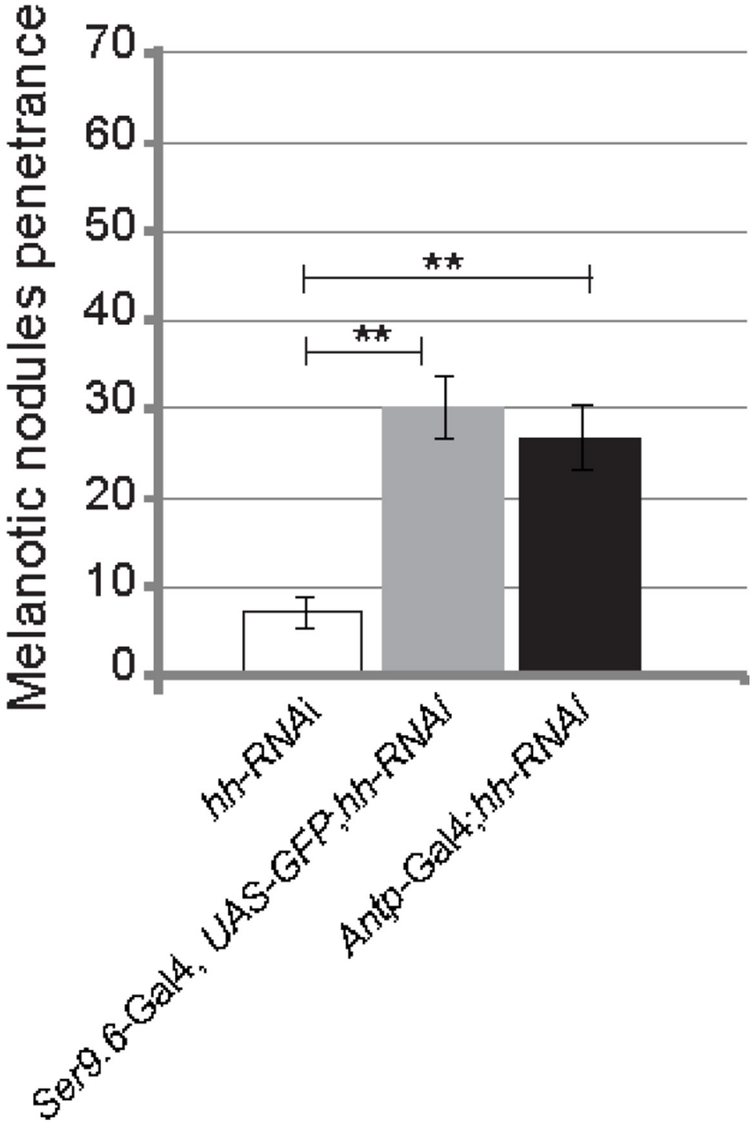

Supplementary Figure S3: *hh*-RNAi expressed in the PSC induces development of melanotic nodules. Penetrance of the melanotic nodule phenotype after expression of *hh*-RNAi in the PSC under the control of either the *Serrate9.6*-Gal4 (gray column, *Ser9.6*-Gal4) or the *Antennapedia*-Gal4 (black column, Antp-Gal4) drivers. The white column indicates the phenotype penetrance in larvae carrying 2 copies of the transgene *UAS-hh-RNAi* (*hh*-RNAi) but no Gal4 driver (negative control). The average phenotype penetrance is calculated from three independents experiments each one based on the analysis of 15–30 larvae. $^{**}P < 0.001$. Error bars indicate s.e.

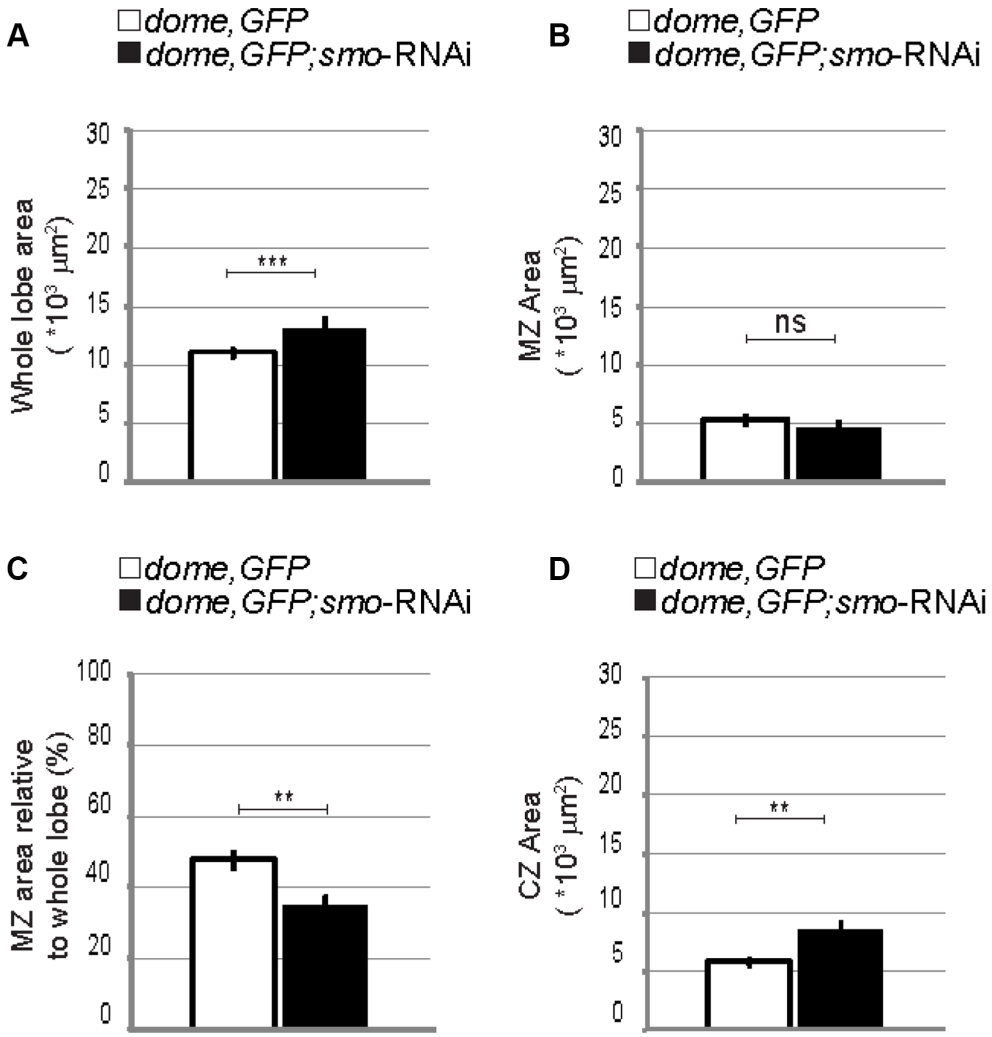

Supplementary Figure S4: Expression of *smo*-RNAi in the MZ of the lymph gland induces loss of hematopoietic precursors. Quantification of the average total primary lobe area (**A**), absolute MZ area (**B**), ratio between MZ area/total area (**C**), CZ area (**D**) in *domeless-Gal4, UAS-GFP/+; UAS-smoRNAi/+* m-L3 larvae (*dome, GFP; smo-RNA*i). The average size of the lobes is increased likely due to increase of the CZ at the expense of the MZ (A, C, D), although the size of the GFP expressing domain is unchanged (B). White columns correspond to control lobes, dissected from larvae carrying only the Gal4 driver (control, *domeless-Gal4, GFP* indicated as *dome, GFP*). The data describe the analysis of 6–10 lobes from 5–8 animals. (***p* <0.001, ****p* <0.0001, ns = not significant). Error bars indicate s.e.

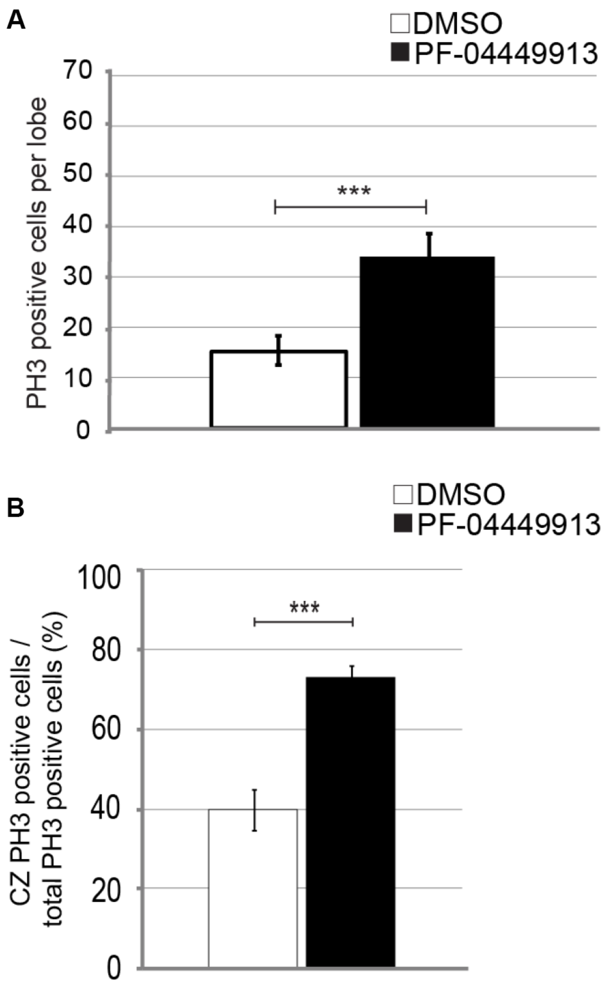

Supplementary Figure S5: PF-04449913 administration induces increase of cell proliferation in the CZ of the lymph gland primary lobe. Quantification of the proliferating cells in lymph gland primary lobes of *hemolectin-Gal4, UAS-GFP* larvae fed with either PF-04449913- or DMSO-medium. The cells immuno-labeled with the anti-PH3 antibody in the CZ (GFP^+ region) and in the MZ (GFP^- region) have been counted in 15 primary lobes of the two groups of larvae. (**A**) Quantification of the average number of $PH3^+$ cells in the whole primary lobe. (**B**) Average ratio between PH3+ cells in the CZ (GFP+) and the total $PH3^+$ cells per lobe. (**p <0.001, ***p < 0.0001). Error bars indicate s.e.
